# Investigation of the Influence of Artificial Intelligence Markup Language-Based LINE ChatBot in Contextual English Learning

**DOI:** 10.3389/fpsyg.2022.785752

**Published:** 2022-04-07

**Authors:** Yu-Cheng Chien, Ting-Ting Wu, Chia-Hung Lai, Yueh-Min Huang

**Affiliations:** ^1^Department of Engineering Science, National Cheng Kung University, Tainan, Taiwan; ^2^Graduate School of Technological and Vocational Education, National Yunlin University of Science and Technology, Yunlin, Taiwan

**Keywords:** LINE ChatBot, artificial intelligence markup language (AIML), competitive strategy, English learning, contextual learning

## Abstract

This study is intended to create an innovative contextual English learning environment making use of the widely used communication software, LINE ChatBot, based on the Artificial Intelligence Markup Language (AIML), in order to improve speaking and listening ability among learners. A total of 73 students were invited to participate in learning activities involving a 4-week English conversation exercise including both speaking and listening. Additionally, in order to explore the influence of competition on language acquisition, we added competition characteristics to the learning activities in the experimental group to enhance learning motivation and learning outcomes. The results showed that with the help of the LINE ChatBot contextual learning environment, the performance of both groups of students was slightly enhanced, but no significant differences were found. Meanwhile, extrinsic motivation in both the experimental and control group was improved if they spoke anonymously. That is, the contextual learning environment based on the LINE ChatBot significantly improved the learners’ English speaking and listening ability. In addition, the results showed that the addition of a competition element effectively enhanced the learners’ intrinsic motivation to learn English on the LINE ChatBot.

## Introduction

In many non-English speaking countries, English acquisition has become an important educational strategy and policy, where the cultivation of ability is dependent on the balanced development of listening, speaking, reading, and writing (Newton et al., 2018). Among these, listening and speaking are considered to be the cores of language interaction, where frequent use of the second language for exchange or communication is conducive to its acquisition (Fang and Baker, 2018). However, it is indeed difficult and stressful for learners who regard English as a second language (ESL) or as a foreign language (EFL) to learn it (Rintaningrum, 2018). Learners may lack self-confidence (Gumartifa and Syahri, 2021) or may reside in non-English speaking areas and thus may be less likely to communicate and talk with others in English (Shadiev et al., 2016). It is therefore important to develop English learning situations that will increase opportunities to engage in conversations in English, which will in turn increase learning motivation and interest (Shadiev et al., 2016).

The advent of new information and communication technology (ICT) has changed how people interact. ICT is also considered to be an effective, practical tool for educational reform (Gilakjani, 2017). Integrating ICT into a learning environment may help learners discuss and reflect and also may help them complete learning tasks (Aşık et al., 2020). In addition, Huang and Hong (2016) maintained that ICT as a learning tool helps people learn various languages and encourages personalized English learning. Mobile-Assisted Language Learning (MALL) is characterized by portability, interactivity, connectivity, and privacy and is carried out in real time. Many studies thus have applied it to the process of learning English to create appropriate contextual learning scenarios (Wu et al., 2011; Panagiotis and Krystalli, 2020). These studies illustrate that mobile technology can help learners extend English acquisition to familiar environments outside the classroom, as well as presenting other contextual learning situations. Ko and Lim (2021) argued that contextual learning may increase both learning resources and scenarios intended to promote personalized English acquisition.

As a matter of fact, we believe that in terms of English conversations, learners in Taiwan, apart from lacking interest in learning, mainly lack opportunities to communicate in English and are thus shy about communicating with others in English. As such, this study is an attempt to make English conversational exercises more interesting through the use of communication technology utilizing mobile devices. Yang et al. (2020) believed that “competition” with a game element can be integrated into English education, where integrating competition into learning activities helps encourage learners to participate more actively in learning activities (Vandercruysse et al., 2013). Consequently, in this study, conversation exercises were carried out with the help of LINE ChatBot communication software, which was designed by LINE and anonymous students. Meanwhile, we added a competition feature into the learning activities to examine the impact of competition on learning outcomes and motivation. The research questions are as follows:

• What are the possible influences of LINE ChatBot on English speaking and listening?
• How does the use of LINE ChatBot with competition for English conversation exercises affect learning motivation?

## Literature Review

For learners, the traditional English teaching environment is often perceived as boring and is used to carry out what they perceive to be unmotivating learning activities (Chen and Chung, 2008), which has decreased learner interest and confidence related to learning, in turn compromising learning effectiveness. This phenomenon reveals a lack of motivation toward learning English for most learners, especially in Asia (Shadiev et al., 2016). Taking Taiwan as an example, the traditional English learning environment is teacher-centered, where teachers mainly focus on vocabulary, grammar, translation, and testing, which leads to few opportunities for students to communicate with each other in English (Chung and Huang, 2010; Chang and Goswami, 2011). Therefore, a variety of different technologies is needed to assist with the English learning process.

Mobile Assisted Language Learning (MALL) is an effective form of learning than traditional lecture-based classrooms and is well-documented in K-12 and higher education (Botero et al., 2018; Cakmak, 2019). It utilizes mobile-related technologies such as smartphones and tablets to support communication in the target language individually or in collaboration with peers in the learning environment (Kukulska-Hulme, 2012). The environment can provide characteristics that include permanency, accessibility, immediacy, interactivity, and contexts for learning activities (Ogata and Yano, 2004). Meanwhile, MALL provides learners with opportunities to practice speaking English in a meaningful way, which leads to significantly greater progress in terms of English fluency (Sun et al., 2017). Ebadijalal and Yousofi (2021) also revealed that mobile-assisted learning is effective in terms of improving learners’ oral proficiency, self-confidence, willingness to communicate, risk-taking, engagement, and self-directed performance.

Communication software that supports mobile technology is increasingly attracting users because it provides instant communication on mobile devices through the Internet. A freeware instant messenger application called LINE for smartphones, tablets, and PCs that allows users to create texts, images, video, and audio functions has become popular, particularly in Asian countries (Wikipedia, 2021). With the exception of serving as a personal messenger, LINE has been extended so it can be effectively applied to language teaching (Liu and Wu, 2016; Chang and Lan, 2021). For example, Liu and Wu (2016) designed an English after-class activity based on the LINE app for role-playing aimed toward making it possible for students to speak, write, and interact in English in contextualized scenarios. Chang and Lan (2021) integrated LINE into flipped English learning as a Foreign Language classroom and investigated college Students’ English performance and their perceptions of the experience. In light of the referenced studies, it appears there is a promising future for further exploration of the LINE app an English education context.

## Methods

### Participants

The subjects in this study included second-grade students from two classes in a high school in southern Taiwan. To be specific, a total of 73 students from two classes participated in this experiment. Due to restrictions of class setting factors, this study research subjects could not be randomly assigned, so one class with 37 students was randomly categorized as the experimental group (17 males and 20 females), while the other class with 36 students was the control group (19 males and 17 females).

### Experimental Design

#### Procedure

**FIGURE 1:**
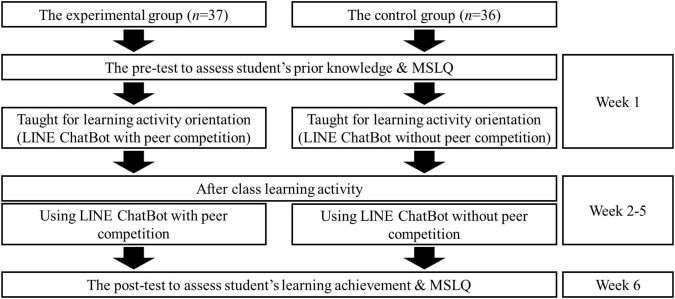
Experimental procedure.

In this study, to control the variables related to the instructors, we invited a Taiwanese English teacher with 5 years of teaching experience to teach the course. In the classroom, the teacher applied the same teaching content and guided learning activities for the two groups of students, and *Studio Classroom* was selected as the materials in this course. Additionally, to understand the effectiveness of the LINE ChatBot with a competition strategy as an English conversational exercise situation, learning activities in this semester were carried out adopting a non-randomized control group and pre-test and post-test quasi-experimental design. The experimental procedure is presented in Figure 1.

Firstly, to ensure the homogeneity of learners’ initial capabilities and behavior, the Students’ listening and speaking ability and learning motivation were tested in a pre-test. *Studio Classroom* learning materials were employed for the listening test, whereas their speaking ability was scored by the instructor using the Multidimensional Fluency Scale (Zutell and Rasinski, 1991). In addition, the Students’ learning motivation was examined using a questionnaire survey in accordance with the Motivated Strategies for Learning Questionnaire (MSLQ) proposed by Pintrich (1991). After completing the pre-test, the instructor explained the learning activities that would take place in this study to the two groups of students, as well as how to use the LINE ChatBot contextual learning environment.

**FIGURE 2:**
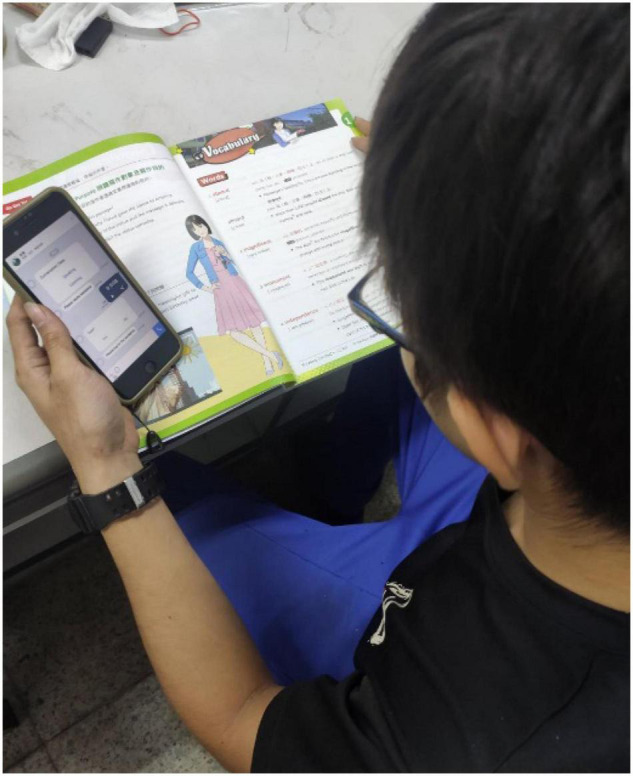
English conversational exercise with speaking and listening in the LINE ChatBot.

In this study, the students in both the experimental and control groups used the LINE ChatBot for English conversation speaking and listening exercises (see Figure 2). The students joined different LINE groups. The students in the control group joined the LINE ChatBot to engage in conversation exercises without competition, whereas those in the experimental group took part in LINE ChatBot conversation exercises that incorporated competition. Both groups of students used the LINE ChatBot for 4 weeks of after-school learning activities. They made use of what they had learned in class, independently selected appropriate sentences from teaching materials as topics for conversation, read aloud their sentences in the LINE group, and listened to the sentences read by their peers as conversation exercises. Notably, the students remained anonymous during the interactions, that is, the students did not identify their peers in the LINE group.

After completion of 4 weeks of after-school learning activities, the students were asked to complete the post-test questions to measure their English listening and speaking ability in an effort to determine the learning effects when using the LINE ChatBot. Meanwhile, in order to investigate the impact of competition on learner motivation, a post-test was also carried out via MSLQ.

#### Learning Activities

In this study, the LINE ChatBot was designed for a learning activity incorporating English conversation, which was based on Artificial Intelligence Markup Language (AIML) to create a contextual English learning environment for the purpose of practicing conversation. Meanwhile, we combined speech-to-text recognition (STR) technology to identify the Student’s conversation. Initially, all of the participants were asked to join a LINE group and to interact with the LINE ChatBot to enhance their speaking and listening abilities. The students in the experimental and control groups were asked to join different groups, the former with the competition element, and the latter without.

**FIGURE 3:**
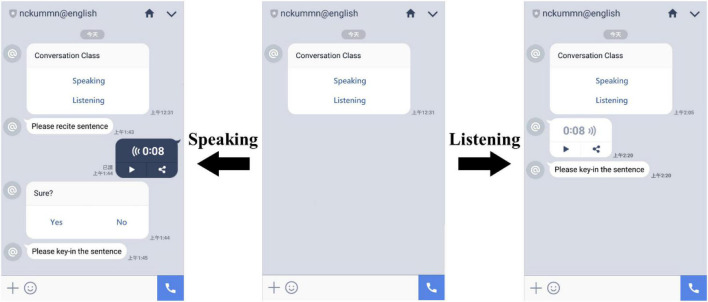
LINE ChatBot contextual learning environment.

After all the students joined the group, the LINE ChatBot automatically asked a member to engage in a role-playing conversation. One student was asked to select a sentence from the classroom learning materials to read aloud in LINE and to input the sentence to complete a speaking exercise in the LINE ChatBot. Subsequently, the other students could engage in listening exercises and give answers via the LINE ChatBot, and those who finished answering could then see the correct responses (see Figure 3). The two groups of students used the LINE ChatBot to carry out a 4-week learning activity. After completing the learning activities each week, all of the students engaged in the conversation exercises, which included one speaking activity as well as 36 listening exercises in the experimental group and 35 exercises in the control group. The classroom teacher would then give feedback to the students on elements of their speaking performance, such as articulation, pronunciation, and tone. Students in the experimental group received additional scores related to the competition phase of the experiment.

### Competition Strategy

The listening and speaking scoring criteria in the LINE ChatBot conversation exercise group was also used for the students in the experimental group. The LINE ChatBot used by the group automatically calculated the Students’ scores based on the criteria. Also, to increase motivation for the competition, the LINE ChatBot informed the group members that the conversation scores would be made according to both the correctness and speed of their responses. After all the students completed the weekly learning activities, the LINE ChatBot announced the weekly scores and rankings.

#### Scoring Criteria for Listening in the Competitive

With respect to listening, learners answered according to the sentences heard in the LINE ChatBot, and then the system would automatically compare the answer input by the learners with the correct answer to acquire the score for correct answers. That is, when the learners input a complete correct sentence, they received 10 points for the correctness score. In the case of spelling errors, 1 point was deducted for each misspelled word. Additionally, the answer speed was scored based on the speed of the responses, where a quicker response led to a higher score. The scoring criteria for listening were based on the following equation, where *w* represents the number of wrong words; *r* represents the answer sequence, and *N* represents the number of members in the group.


$$listeningscore=\left(10-w\right)+\left(1-\frac{r}{N-1}\right)$$

#### Scoring Criteria for Speaking in the Competitive

For the speaking score, to enable learners to practice more accurately based on their articulation and pronunciation, the scoring criteria included the respondents’ scores in the calculation. In other words, after all the students completed the listening exercise and obtained their scores, the LINE ChatBot took the average listening score of the entire class as the learners’ speaking score. Therefore, the scoring criteria for speaking in the LINE ChatBot were based on the following equation.


$$\mathrm{speaking}\mathrm{score}=\sum_{n=1}^{N-1}\frac{{\left(10-w\right)}_{n}}{N-1}$$

## Results

### The ANCOVA for Speaking and Listening Abilities

The two groups of students engaged in contextual learning via the LINE ChatBot to determine whether there were significant differences in their English speaking and listening performance. In the statistical analysis, a single-factor ANCOVA was employed to explore whether there were significant differences in learning effectiveness with or without competition in the learning intervention. Table 1 shows the statistics for the pre-test and post-test for speaking and listening with and without a competition element.

**TABLE 1:** Pre- and post-test descriptive statistics for the speaking and listening performance.

		Experimental group (With competition)			Control group (Without competition)			
		*n*	*Mean*	*SD*	*n*	*Mean*	*SD*	*F*
Listening	Pre-test	37	9.54	2.22	36	9.47	2.26	0.143
	Post-test	37	10.42	2.29	36	10.08	2.23	
Speaking	Pre-test	37	62.08	18.75	36	62.80	18.71	0.808
	Post-test	37	64.70	16.66	36	64.83	17.51	
								

According to Table 1, the average speaking ability post-test scores for the two groups of students were higher than the average pre-test scores, and the post-test scores for listening ability were also higher than the pre-test scores. An ANCOVA was used to verify if the scores for the experimental and control groups were statistically significantly different. The test results for intra-group regression coefficient homogeneity showed that speaking and listening ability was *F* = 0.128 (*p* > 0.05) and *F* = 0.875 (*p* > 0.05), respectively, which failed to reach significance. Therefore, the results indicated that the slopes of the two groups of regression lines were the same, proving the intra-group regression coefficient homogeneity of the covariates, so the ANCOVA could thus be conducted. After the impact of pre-test effectiveness was eliminated, the results of the ANCOVA showed that the scores for speaking ability were *F* = 0.143 (*p* > 0.05), and those for listening ability were *F* = 0.808 (*p* > 0.05), both failing to reach significance. The results suggest that there were no between-group differences in the results of the post-test for the two groups of students who learned with and without competition. The results thus indicated that when the students engaged in contextual learning, learning with or without competition did not affect their speaking and listening abilities.

### ANCOVA for Intrinsic and Extrinsic Learning Motivation

To gain a more in-depth understanding of whether learning with or without a competitive strategy affected the Students’ learning motivation in the LINE ChatBot contextual learning environment, a pre-test and a post-test were performed with intrinsic and extrinsic goal orientation in MSLQ, which included four items measured using a Likert 7-point scale. In order to present a comparison of the Students’ learning motivation, the pre-test and post-test results for learning motivation are listed in Table 2 with and without competition.

**TABLE 2:** Pre- and post-test descriptive statistics for intrinsic/extrinsic motivation.

		Experimental group (With competition)			Control group (Without competition)			
		*n*	*Mean*	*SD*	*n*	*Mean*	*SD*	*F*
Intrinsic	Pre-test	37	17.08	3.63	36	16.50	3.47	22.85***
	Post-test	37	19.35	2.53	36	17.22	3.01	
Extrinsic	Pre-test	37	17.30	3.51	36	16.94	3.32	0.303
	Post-test	37	18.89	2.88	36	19.19	2.59	

***p < 0.001.

In Table 2, the average post-test scores for intrinsic and extrinsic learning motivation for both groups of students were higher than the average pre-test scores. Firstly, the test results for the intra-group regression coefficient homogeneity showed that the scores for extrinsic motivation were *F* = 3.016 (*p* > 0.05), and those for intrinsic motivation were *F* = 3.232 (*p* > 0.05), which failed to reach significance and indicated intra-group homogeneity. After the impacts caused by pre-test effectiveness was eliminated, an ANCOVA statistical analysis was conducted, the scores for intrinsic motivation were *F* = 22.85 (*p* < 0.001), which reached significance, while those of extrinsic motivation were *F* = 0.303 (*p* > 0.05), which failed to reach significance. The results indicated that with respect to the Students’ learning motivation during the process of learning with or without competition, there were significant differences in intrinsic motivation. However, there were no significant differences in extrinsic motivation. Furthermore, after the impact of the pre-test results was eliminated, the marginal means of the post-test scores for intrinsic and extrinsic motivation after adjustment to the experimental group were 19.37 and 18.91, respectively. Those of the post-test scores for intrinsic and extrinsic motivation after adjustment to the control group were 17.21 and 19.18, respectively. The results indicated that when students engaged in contextual learning, learning with or without competition affected their intrinsic learning motivation.

## Discussion

This study was an investigation of English conversation exercises utilizing the LINE ChatBot communication software. According to the findings, the post-test of speaking and listening scores in the experimental and control groups were higher than those obtained in the pre-test. This finding was in line with the results of previous related studies using communication software. For example, Liu and Wu (2016) found that practicing English using the LINE app can enhance speaking performance. Cetinkaya and Sütçü (2018) also stated that the Facebook and WhatsAPP ICT enhances Students’ speaking and listening ability. Using technology support, Students’ speaking ability can be enhanced through practice (Shadiev and Yang, 2020). Krebt (2017) also argued that listening ability can be enhanced by listening to peers. Hwang et al. (2016) suggested that listening and speaking ability can be improved with practice. Above all, speaking and listening practice can improve English acquisition performance. A previous study showed that the pronunciation of low-achieving English students who learned with the pop song improved (Kim and Kang, 2015).

The learning performance was not found to be affected by a competitive environment in the learning process via the LINE ChatBot since both groups had an equal opportunity to train their speaking and listening. Therefore, the difference in terms of learning ability failed to reach a significant level. However, as for learning motivation, intrinsic motivation was increased by competition, especially achievement motivation (Chen, 2019; Liu, 2020). When the students focused on their rank in the competition, it motivated them to win the competition. Furthermore, with an anonymous login, students could overcome their shyness related to speaking, which may have enhanced their extrinsic motivation (Wallace, 2016). This improvement was also attributed to the reward for winning the competition. Deci and Ryan (1985) revealed that a reward has an impact on behavioral control and enhances interest in a task. It is a reasonable to infer that the students were motivated to strive to achieve a higher rank when watching their ranking scores. In addition, since the mechanism provided by the LINE ChatBot prevents students from recognizing who provided the pronunciation being heard, the students were encouraged to concentrate more on their English listening and speaking and less on who was speaking.

## Conclusion

In general, in countries where English is not the mother tongue, opportunities to communicate in English are very limited, in turn contributing to poor conversational ability. In Taiwan, teachers are generally excessively dependent on traditional teaching methods, that is, concentrating on English reading and writing performance while ignoring the fact that language should focus on conversational communication. Thus, this study uses an innovative contextual English learning environment so that learners could anonymously and confidently recite the sentences in the teaching materials and listen to their peers speak in order to enhance their conversational ability in English. More importantly, the contextual learning environment proposed in this study is not limited to classroom teaching and can be integrated into the surrounding environment. Although the findings of this study confirmed the effectiveness of the LINE ChatBot for developing English speaking and listening skills, this study has several limitations: Firstly, the participants in this study were located in southern Taiwan. The group assignments were random, but not all high school students were included. The second limitation is the competition strategy used in the learning procedure. This study did not discuss other types of competition, such as one-on-one competitions or group competitions. Finally, we implemented the learning procedure with the LINE ChatBot, but we didn’t consider other platforms that might have different effects on conversations in English or have different exercises.

Overall, using the LINE ChatBot as an after-school contextual learning environment for English conversation exercises that include both speaking and listening, this study also explored the impact of competition on learners. The experimental results indicated that competition led to no significant between-group differences in terms of learning effectiveness, but the speaking and listening ability in the two groups was slightly improved, showing that the contextual learning environment proposed herein enhance their conversational ability in English. In addition, with respect to learning motivation, we found that the level of extrinsic motivation in the experimental group was significantly higher than that of the control group. This is because competition can boost learners’ interest or level of pleasure in an activity based on the rankings.

As a result, use of the LINE ChatBot has several benefits. First, mobile technology greatly facilitates language acquisition, allowing people to use it for conversation exercises at any time and any place. Second, communication using human voices replaces language learning using machine pronunciation, with the former being more intimate and appealing. Finally, conversation exercises that are carried out anonymously can effectively reduce the negative impact of shyness when communicating in English.

## Data Availability Statement

The original contributions presented in the study are included in the article/supplementary material, further inquiries can be directed to the corresponding author/s.

## Ethics Statement

Written informed consent was obtained from the individual(s) for the publication of any potentially identifiable images or data included in this article.

## Author Contributions

Y-CC, T-TW, and Y-MH contributed to the conception and design of the study. T-TW and C-HL organized the database and performed the statistical analysis. Y-CC, C-HL, and Y-MH wrote the first draft of the manuscript. T-TW wrote sections of the manuscript. T-TW and Y-MH managed supervision, project administration, and funding acquisition. All authors contributed to manuscript revision, read, and approved the submitted version.

## Conflict of Interest

The authors declare that the research was conducted in the absence of any commercial or financial relationships that could be construed as a potential conflict of interest.

## Publisher’s Note

All claims expressed in this article are solely those of the authors and do not necessarily represent those of their affiliated organizations, or those of the publisher, the editors and the reviewers. Any product that may be evaluated in this article, or claim that may be made by its manufacturer, is not guaranteed or endorsed by the publisher.
